# SERMs (selective estrogen receptor modulator), acting as estrogen receptor β agonists in hepatocellular carcinoma cells, inhibit the transforming growth factor-α-induced migration via specific inhibition of AKT signaling pathway

**DOI:** 10.1371/journal.pone.0262485

**Published:** 2022-01-10

**Authors:** Rie Matsushima-Nishiwaki, Noriko Yamada, Yuria Hattori, Yui Hosokawa, Junko Tachi, Takamitsu Hori, Osamu Kozawa

**Affiliations:** Department of Pharmacology, Gifu University Graduate School of Medicine, Gifu, Japan; Duke University School of Medicine, UNITED STATES

## Abstract

Selective estrogen receptor modulator (SERM) interacts with estrogen receptors and acts as both an agonist or an antagonist, depending on the target tissue. SERM is widely used as a safer hormone replacement therapeutic medicine for postmenopausal osteoporosis. Regarding hepatocellular carcinoma (HCC), accumulating evidence indicates gender differences in the development, and that men are at higher morbidity risk than premenopausal women, suggesting that estrogen protects against HCC. However, it remains unclear whether SERM affects the HCC progression. Previously, we have shown that transforming growth factor (TGF)-α promotes the migration of HCC cells via p38 mitogen-activated protein kinases (MAPK), c-*Jun* N-terminal kinase and AKT. In the present study, we investigated whether SERM such as tamoxifen, raloxifene and bazedoxifene, affects the HCC cell migration using human HCC-derived HuH7 cells. Raloxifene and bazedoxifene but not tamoxifen, significantly suppressed the TGF-α-induced HuH7 cell migration. ERB041 and DPN, estrogen receptor (ER) β agonists, inhibited the TGF-α-induced cell migration whereas PPT, an ERα agonist, did not show the suppressive effect on the cell migration. ERB041 attenuated the TGF-α-induced phosphorylation of AKT without affecting the phosphorylation of p38 MAPK and *c-Jun* N-terminal kinase. Raloxifene and bazedoxifene also inhibited the phosphorylation of AKT by TGF-α. Furthermore, PHTPP, an ERβ antagonist, significantly reversed the suppression by both raloxifene and bazedoxifene of the TGF-α-induced cell migration. Taken together, our results strongly indicate that raloxifene and bazedoxifene, SERMs, suppress the TGF-α-induced migration of HCC cells through ERβ-mediated inhibition of the AKT signaling pathway.

## Introduction

Selective estrogen receptor modulator (SERM) belongs to a class of compounds with non-steroidal structures that interact with estrogen receptors (ERs), ERα and ERβ [1–4]. Estrogen has agonistic effects on various tissues especially breast and uterus, and the long term usage of estrogen increases the risk of breast and uterine cancer [1]. On the other hand, SERM acts as either agonist or antagonist of ER in a tissue-specific manner [1–4]. The tissue-specific expression of ERα and ERβ, and conformations of ER dimerization induced by interaction of SERM are speculated to affect the tissue-specific activity of SERM [1, 2]. Accumulating evidence indicates that expression of tissue-specific co-regulators (co-activators and co-repressors) and their interaction with the ER dimer also modulate the tissue specificity of SERM [1, 2]. Currently, SERM is classified into three generations [1–4]. Tamoxifen, a SERM of the first-generation, which was developed as an ER antagonist for breast cancer treatment, plays as an agonist of ER in bone tissue and upregulates bone mass [1–4]. In addition, tamoxifen acts as an agonist of ER in uterus and increases the risk of endometrial cancer [1–4]. The second-generation SERM, raloxifene, exhibits different tissue specificities compared with tamoxifen [1, 2]. Raloxifene that has been initially developed for breast cancer therapy, reduces the incidence of osteoporosis in postmenopausal women, indicating that raloxifene functions as an ER agonist in bone [1, 2]. Further, raloxifene shows the preventive effect on endometrial cancer [1, 2]. Bazedoxifene, a third-generation SERM, displays an agonistic effect on ER in bone and an antagonistic effect on ER in breast and endometrium [2]. Especially, in endometrium, bazedoxifene shows more strong antagonistic effect compared with raloxifene [2, 4]. Currently, raloxifene and bazedoxifene are widely used as safer medicines for patients with osteoporosis as postmenopausal hormone replacement therapy [1–4].

Primary liver cancer is the third leading cause of cancer-related death globally, with nearly 80% or more of it being hepatocellular carcinoma (HCC) [5, 6]. Chronic infections with hepatitis B virus (HBV) and hepatitis C virus (HCV) are the highest risk factors for HCC [5, 6]. However, in developed countries, excess body weight and diabetes increase as risk factors for HCC [5, 6]. High mortality of HCC is due to the high rate of recurrence and metastasis even after surgical removal of tumors or liver transplantation [5, 7–9]. Circulating HCC tumor cells and HCC-derived exosomes are the cause of recurrence and metastasis [7, 9]. There are gender differences in HCC. HCC develops higher in men than in women, especially premenopausal women, both in incidence and mortality, and men have been significantly younger than women at diagnosis [6, 10, 11]. In addition, epidemiological studies suggested that female patients have fewer vascular invasion and bone metastasis of HCC cells than male patients [12, 13]. Menopause hormone therapy with estrogen reportedly reduces the risk of HCC and the increased overall survival times of HCC patients [14]. Regarding ER in HCC cells, ERα and ERβ are expressed in HCC, but expression levels of both ERs, especially ERα, are decreased in tumors compared with those in non-tumorous liver tissue [15, 16]. In addition, the ERβ expression levels have been reported to be inversely correlated with HCC progression [17]. However, the exact roles of ERα and ERβ in HCC cell function, especially cell migration remain to be clarified.

Mounting evidence indicates that dysregulation of growth factors/growth factor receptors signaling pathway is implicated in HCC progression, including HCC metastasis [18, 19]. Among growth factors, it is currently recognized that transforming growth factor-α (TGF-α) is overexpressed in HCC and mainly contributes to HCC cell invasion [18–21]. Previously, we have reported that TGF-α induces the migration of human HCC-derived HuH7 cells, and that p38 mitogen-activated protein kinase (MAPK), c*-Jun* N-terminal kinase (JNK) and AKT play as positive regulators in the migration [22–24]. In the present study, we investigated the effects of tamoxifen, raloxifene or bazedoxifene on the TGF-α-induced migration of HCC-derived HuH7 cells. We here demonstrate that raloxifene and bazedoxifene, but not tamoxifen, suppressed TGF-α-induced migration of HuH7 cells, and that the suppressive effects were exerted through ERβ-mediated inhibition of AKT.

## Materials and methods

### Materials

Recombinant human TGF-α was purchased from R&D Systems, Inc. (Minneapolis, MN). Tamoxifen, raloxifene hydrochloride (raloxifene), and bazedoxifene acetate (bazedoxifene) were obtained from Sigma-Aldrich, Co. (St. Louis, MO). ERB041 was obtained from Tocris Bioscience, Co. (Bristol, UK). Propylpyrazole triol (PPT), 2,3-bis(4-hydroxyphenyl)propionitrile (DPN), 4-[2-phenyl-5,7-bis(trifluoromethyl)pyrazolo[1,5-a]pyrimidin-3-yl]-phenol (PHTPP) and G-1 were obtained from Cayman Chemical Co. (Ann Arbor, MI). NSC23766 was purchased from Tocris Bioscience (Bristol, UK). Lactacystin was obtained from Calbiochem, Inc. (San Diego, CA). Phospho-specific epidermal growth factor receptor (EGFR) antibodies (#2234), phospho-specific p38 MAPK antibodies (#4511), phospho-specific c*-Jun* N-terminal kinase (JNK) antibodies (#4668), phospho-specific AKT antibodies (#13038) were purchased from Cell Signaling Technology, Inc. (Danvers, MA). Glyceraldehyde-3-phosphate dehydrogenase (GAPDH) antibodies (sc47724) were purchased from Santa Cruz Biothechnology, Inc. (Dallas, TX). Other chemicals were obtained from FUJIFILM Wako Pure Chemical, Co. (Osaka, Japan). A Rac1 Activation Assay Kit was obtained from Millipore Co. (Billerica, MA). Other materials were purchased from commercial sources. Tamoxifen, raloxifene, bazedoxifene, PPT, ERB041, DPN, and PHTPP were dissolved in dimethyl sulfoxide (DMSO). The maximum DMSO concentration (0.1%) did not affect cell migration assay or Western blot analysis.

### Cell culture

Human HCC-derived HuH7 cells (JCRB0403) were obtained from the JCRB Cell Bank (Tokyo, Japan) [25]. The cultured HuH7 cells were maintained in Roswell Park Memorial Institute (RPMI) 1640 medium (Sigma-Aldrich Co.) containing 10% of fetal calf serum (FCS; Hyclone Laboratories Inc., Logan, UT). For cell migration assay, the cultured cells were seeded into100-mm diameter dishes (4 × 10^5^ cells/dish), cultured for 4 days, and then used for the assay. In the case of Western blotting and analysis of Rac activation, the HuH7 cells were seeded into 100-mm diameter dishes (6 × 10^5^ cells/dish). After 3 days, the cultured medium was replaced with the serum-free medium, and the cells were then used after 24 h.

### Cell migration assay

A transwell cell migration assay was performed using a Boyden chamber (polycarbonate membrane with 8-μm pores, Transwell; Costrer; Corning, Inc., Corning, NY) as described previously [22]. In brief, the cultured cells were seeded in the upper chamber (1 × 10^5^ cells/well) with the serum-free medium. The cells were then pretreated with tamoxifen, raloxifene, bazedoxifene, PPT, ERB041, DPN or NSC23766 in the upper chamber for 60 min at 37°C. After pretreatment, TGF-α was added to the lower chamber and incubated at 37°C for 23 h. When indicated, the cells were exposed with PHTPP for 60 min in the upper chamber before the treatment of ERB041, raloxifene or bazedoxifene. After the incubation, un-migrated cells on the upper surface of the membrane at the bottom of the upper chamber were mechanically removed using cotton swabs. The migrated cells adherent to the under-surface of the membrane were fixed with 4% paraformaldehyde (Alfa Aesar, Thermo Fisher Scientific Co., Lancashire, UK), and then stained with 4’,6-diamino-2-phenylindole (DAPI) solution. The migrated cells were photographed using fluorescent microscopy at a magnification of 20× and counted.

### Western blotting

The cultured cells were pretreated with raloxifene, bazedoxifene, ERB041 or DPN at the indicated concentrations for 8 h. For treatment of G-1, the cells were pretreated with 1 μM of G-1 for 60 min. When indicated, the cells were exposed with lactacystin immediately prior to the treatment with ERB041 or raloxifene. The cells were then stimulated with 30 ng/ml of TGF-α or vehicle at 37°C for 5 min for p38 MAPK, 20 min for JNK, and 1 min for EGFR and AKT. After the stimulation, the cells in each dish were washed with ice-cold phosphate-buffered saline (PBS), and then lysed with the lysis buffer [62.5 mM Tris-HCl (pH6.8), 2% sodium dodecyl sulfate (SDS), 50 mM dithiothreitol, and 10% glycerol]. SDS-polyacrylamide gel electrophoresis (PAGE) and Western blotting were performed as described previously [22] using phospho-specific EGFR antibodies, phospho-specific p38 MAPK antibodies, phospho-specific JNK antibodies, phospho-specific AKT antibodies, and GAPDH antibodies as primary antibodies. Peroxidase-labeled anti IgG antibodies were used for secondary antibodies. The peroxidase activities on a polyvinylidene difluoride membrane (Bio-Rad Laboratories, Inc., Hercules, CA) were detected using ECL Western blotting detection system (Global Life Sciences Solutions Operations, UK Ltd., Buckinghamshire, UK). An image analysis software program (image J, version 1.48; NIH, Bethesda, MD) was used for densitometric analysis. The background-subtracted signal intensity of each phosphorylation signal was normalized with the signal intensity of GAPDH.

### Analysis of Rac activation

The cultured cells were treated with 30 ng/ml of TGF-α for indicated times. For analysis of NSC23766 effect, the cells were pretreated with 300 μM of NSC23766 or vehicle for 60 min, and then stimulated with 30 ng/ml of TGF-α at 37°C for 5 min. After stimulation, the cells in each dish were washed with tris-buffered saline (TBS) and then lysed with Mg^2+^ lysis/wash buffer (MLB). GTP-binding Rac was immunoprecipitated using a Rac1 Activation Assay Kit as described in the manufacturer’s instruction manual. The immunoprecipitated GTP-binding Rac and pre-immunoprecipitated lysates, that is total Rac, were subjected to Western blot analysis using antibodies against Rac.

### Statistical analysis

The data from the experiments are expressed as the mean ± standard deviation (SD). The data were analyzed by an analysis of variance (ANOVA) with Tukey’s post hoc test. The values of *p*<0.05 were considered to be statistically significant.

## Results

### Effects of tamoxifen, raloxifene and bazedoxifene on the TGF-α-induced migration of HuH7 cells

**Fig 1:**
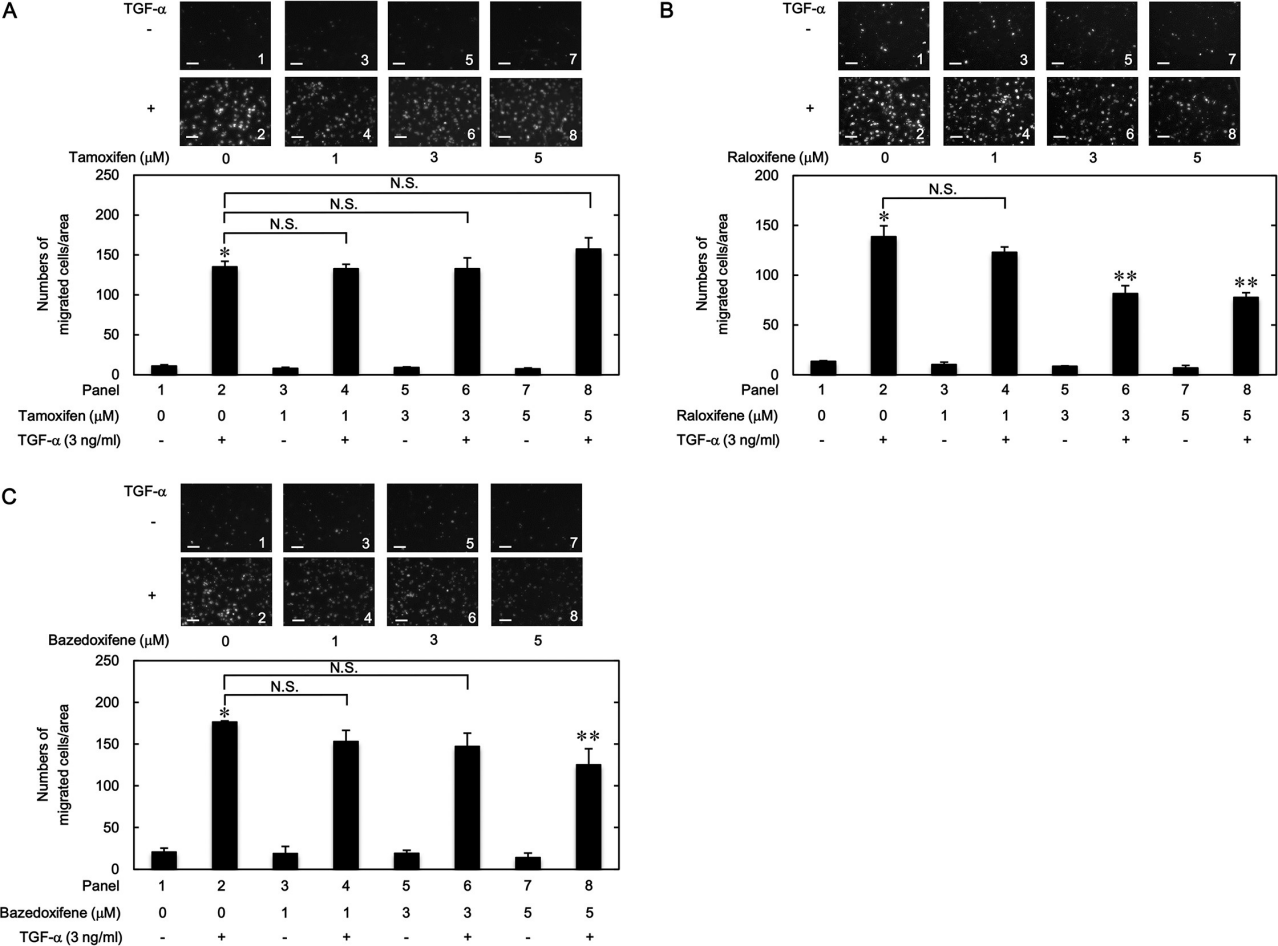
Effects of tamoxifen, raloxifene and bazedoxifene on the TGF-α-induced migration of HuH7 cells. The cells were pretreated with the indicated concentrations of tamoxifen (A), raloxifene (B) or bazedoxifene (C) for 60 min, and then stimulated by 3 ng/ml of TGF-α or vehicle for 23 h. The migrated cells were stained with DAPI for the nuclei. The cells were photographed by fluorescent microscopy at a magnification of 20× (upper panel) and counted (bar graph). Each value represents the mean ± SD of triplicate determinations from three independent cell preparations. *p<0.05, compared to the value of the control cells without TGF-α stimulation (panel 1). **p<0.05, compared to the value of TGF-α stimulation alone (panel 2). N.S. designates no significant difference between the indicated pairs. Scale bar: 100 μm.

Previously, we showed that TGF-α stimulates the migration of human HCC-derived HuH7 cells [22–24]. Thus, at first, we investigated whether tamoxifen, raloxifene and bazedoxifene, representing the three generations of SERMs, affect the TGF-α-induced migration of HuH7 cells. As shown in Fig 1A, tamoxifen had no effect on the migration of HuH7 cells at concentrations up to 5 μM. On the contrary, raloxifene at 3 and 5 μM (Fig 1B), and bazedoxifene at 5 μM (Fig 1C) significantly suppressed the TGF-α-induced migration of HuH7 cells.

#### Effects of PPT, ERB041, DPN and PHTPP on the TGF-α-induced migration of HuH7 cells

**Fig 2:**
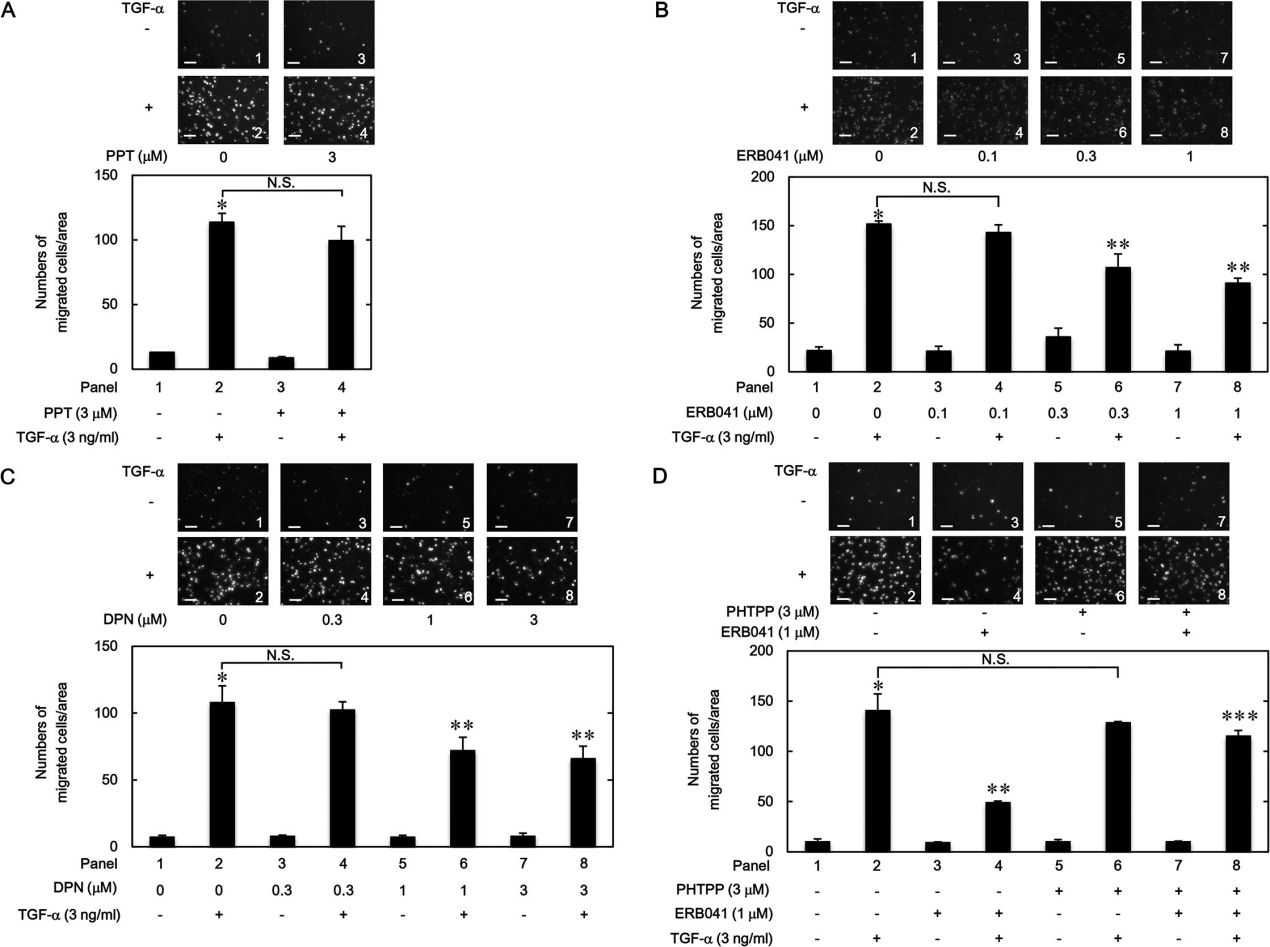
Effects of PPT, ERB041, DPN and PHTPP on the TGF-α-induced migration of HuH7 cells. The cells were pretreated with the indicated concentrations of PPT (A), ERB041 (B and D) or DPN (C) for 60 min, and then stimulated with 3 ng/ml of TGF-α or vehicle for 23 h. When indicated (D), the cells were exposed with 3 μM of PHTPP 60 min prior to the ERB041 treatment. The migrated cells were stained with DAPI for the nuclei. The cells were photographed by fluorescent microscopy at a magnification of 20× (upper panel) and counted (bar graph). Each value represents the mean ± SD of triplicate determinations from three independent cell preparations. *p<0.05, compared to the value of the control cells without TGF-α stimulation (panel 1). **p<0.05, compared to the value of TGF-α stimulation alone (panel 2). ***p<0.05, compared to the value of TGF-α stimulation with ERB041 pretreatment (panel 4). N.S. designates no significant difference between the indicated pairs. Scale bar: 100 μm.

It is generally known that two types of ER are recognized and denoted ERα and ERβ [1–3], and that ERα and ERβ exist in HCC cells [15–17]. Therefore, we next investigated whether ERα or ERβ mediates the suppressive effects by raloxifene and bazedoxifene on the TGF-α-induced migration of HuH7 cells. PPT, an agonist of ERα [26], did not inhibit the TGF-α-induced migration of HuH7 cells even at 3 μM (Fig 2A). On the other hand, ERB041, an ERβ agonist [27], significantly suppressed the HuH7 cell migration by TGF-α at 0.3 and 1 μM (Fig 2B). In addition, DPN, another ERβ agonist [28], also inhibited the migration of HuH7 cells (Fig 2C). In order to further clarify the ERβ-mediated inhibition of the TGF-α-induced migration of HuH7 cells, we examined the effect of PHTPP, an ERβ antagonist [29], on the suppression by ERB041. As shown in Fig 2D, PHTPP, which alone did not affect the HuH7 cell migration by TGF-α, significantly reversed the inhibition by ERB041 of the cell migration.

### Combined effects of PPT and ERB041 on the TGF-α-induced migration of HuH7 cells

**Fig 3:**
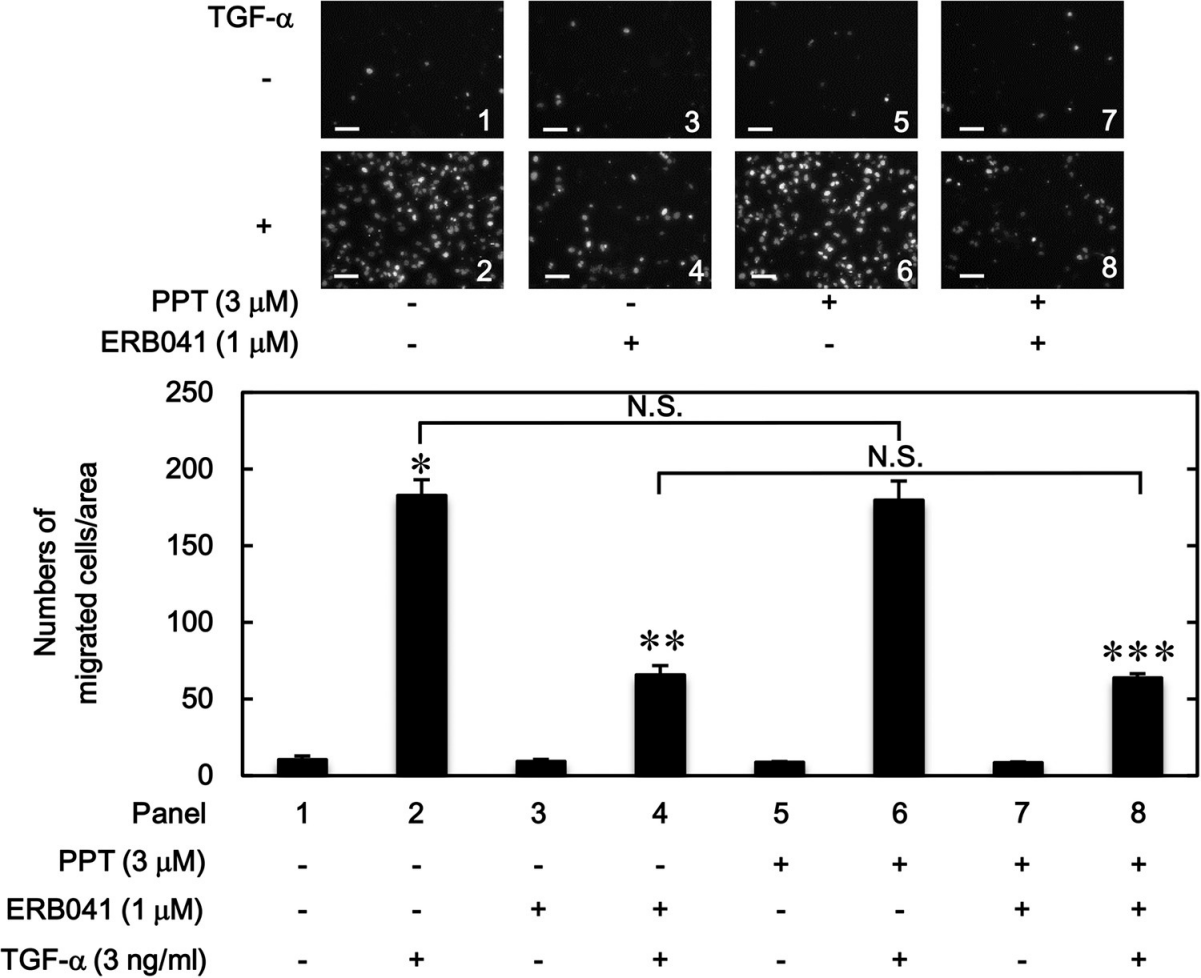
Combined effects of PPT and ERB041 on the TGF-α-induced migration of HuH7 cells. The cells were pretreated with PPT (3 μM) and ERB041 (1 μM) for 60 min, and then stimulated by 3 ng/ml of TGF-α or vehicle for 23 h. The migrated cells were stained with DAPI for the nuclei. The cells were photographed by fluorescent microscopy at a magnification of 20× (upper panel) and counted (bar graph). Each value represents the mean ± SD of triplicate determinations from three independent cell preparations. *p<0.05, compared to the value of the control cells without TGF-α stimulation (panel 1). **p<0.05, compared to the value of TGF-α stimulation alone (panel 2). ***p<0.05, compared to the value of TGF-α stimulation with PPT pretreatment (panel 6). N.S. designates no significant difference between the indicated pairs. Scale bar: 100 μm.

We furthermore elucidated how the simultaneous stimulation of ERα and ERβ affects the TGF-α-induced migration of HuH7 cells. As shown in Fig 3, PPT, an agonist of ERα, affected neither the stimulative effect of TGF-α alone on the migration of HuH7 cells nor the suppressive effect of ERB041, an ERβ agonist, on the TGF-α-induced cell migration.

### Effects of ERB041 on the TGF-α-induced phosphorylation of EGFR, p38 MAPK, JNK and AKT in HuH7 cells

**Fig 4:**
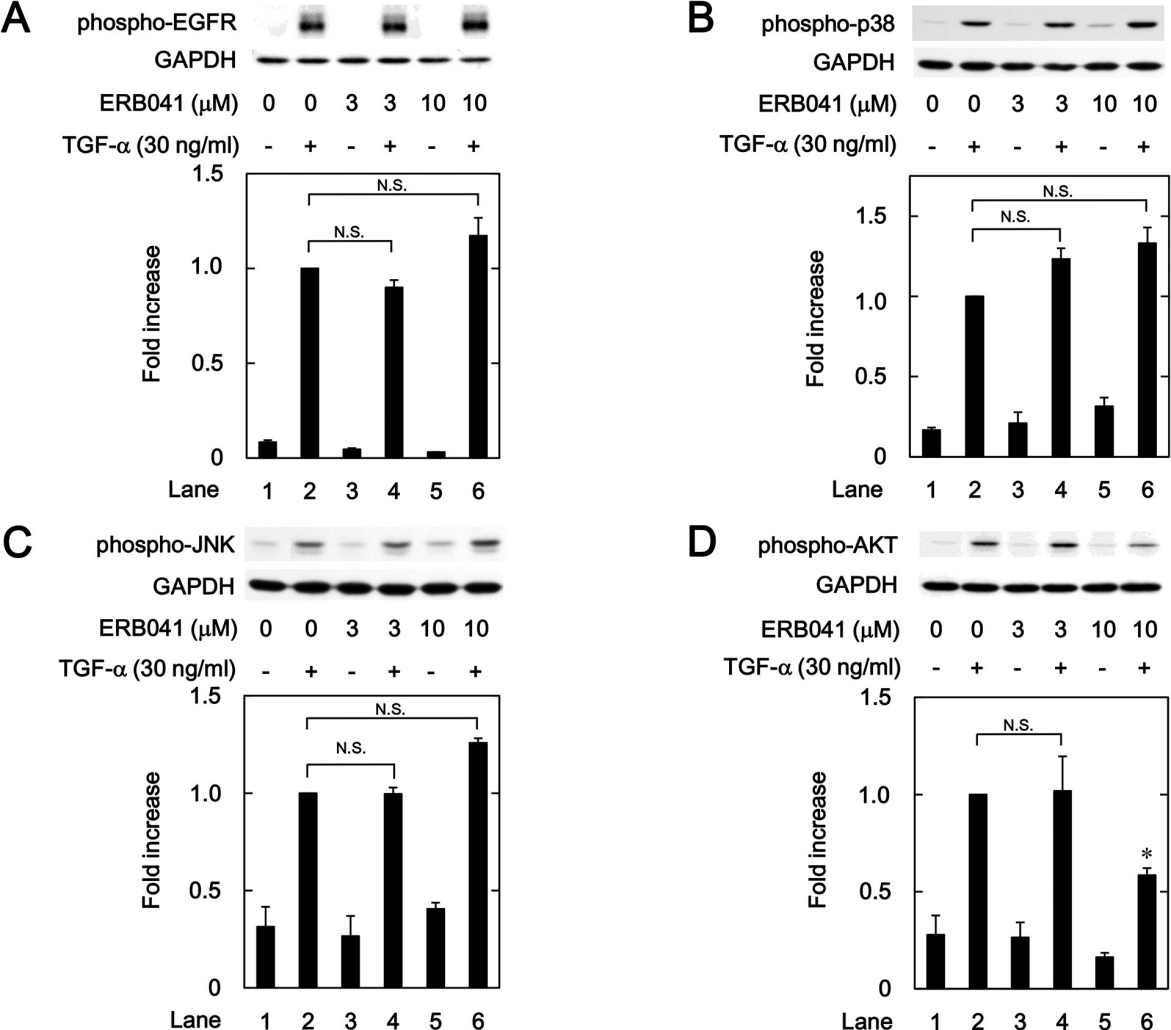
Effects of ERB041 on the TGF-α-induced phosphorylation of EGFR (A), p38 MAPK (B), 54 kDa JNK (C) and AKT (D) in HuH7 cells. The cells were pretreated with the indicated concentrations of ERB041 for 8 h, and then stimulated by 30 ng/ml of TGF-α or vehicle for 1 min for EGFR (A) and AKT (D), 5 min for p38 MAPK (B), or 20 min for JNK (C). The histogram shows the quantitative representation of the phosphorylated levels after normalization with respect to GAPDH obtained from a densitometric analysis. The average of density levels of the TGF-α stimulation alone (lane 2) was expressed as 1.0. Each value represents the mean ± SD of triplicate determinations from three independent cell preparations. *p<0.05, compared to the value of the control cells with TGF-α stimulation alone (lane 2). N.S. designates no significant difference between the indicated pairs.

It is firmly established that TGF-α, a ligand of EGFR, activates the EGFR by inducing auto-phosphorylation, which in turn stimulates many intracellular signaling pathways, such as the MAPKs and AKT pathways [18, 19, 30]. In our previous studies [23, 24], we have demonstrated that TGF-α stimulates the migration of HuH7 cells via activation of p38 MAPK, JNK and AKT. Thus, we next investigated whether the activation of ERβ affects the intracellular signaling pathways in HuH7 cells. As shown in Fig 4A, ERB041, an ERβ agonist, failed to affect the TGF-α-induced auto-phosphorylation of EGFR. In addition, ERB041 did not inhibit the TGF-α-induced phosphorylation of p38 MAPK or JNK (Fig 4B and 4C). However, ERB041 significantly suppressed the TGF-α-induced phosphorylation of AKT (Fig 4D).

### Effects of DPN on the TGF-α-induced phosphorylation of EGFR and AKT in HuH7 cells

**Fig 5:**
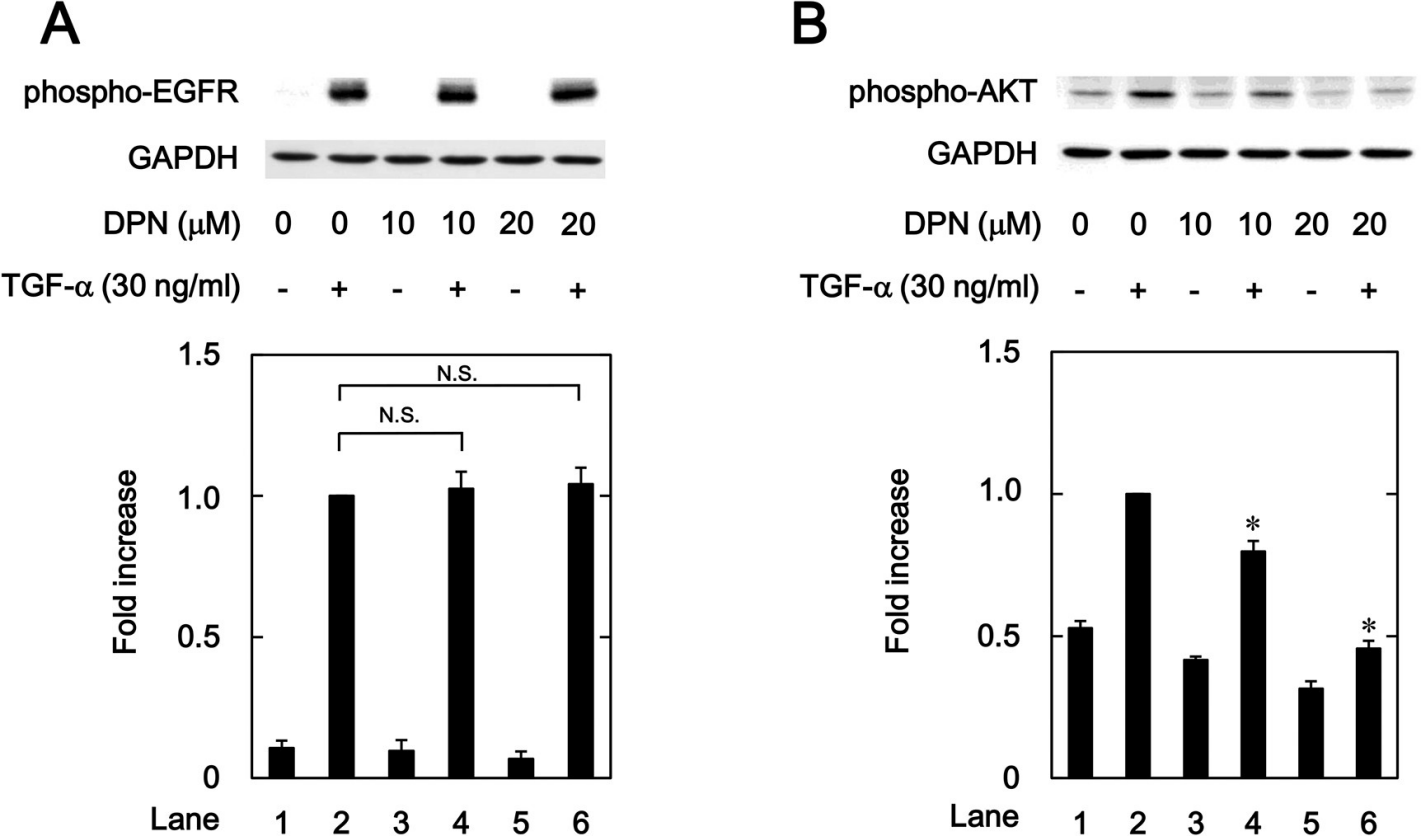
Effects of DPN on the TGF-α-induced phosphorylation of EGFR (A) and AKT (B) in HuH7 cells. The cells were pretreated with the indicated concentrations of DPN for 8 h, and then stimulated by 30 ng/ml of TGF-α or vehicle for 1 min. The histogram shows the quantitative representation of the phosphorylated levels after normalization with respect to GAPDH obtained from a densitometric analysis. The average of density levels of the TGF-α stimulation alone (lane 2) was expressed as 1.0. Each value represents the mean ± SD of triplicate determinations from three independent cell preparations. *p<0.05, compared to the value of the control cells with TGF-α stimulation alone (lane 2). N.S. designates a significant difference between the indicated pairs.

To further investigate the effect of ERβ on the TGF-α-induced activation of AKT in HuH7 cells, we examined the effects of DPN, another ERβ agonist, on the TGF-α-induced phosphorylation of EGFR and AKT. DPN did not affect the auto-phosphorylation of EGFR at concentrations up to 20 μM (Fig 5A). On the other hand, DPN as well as ERB041 significantly attenuated the TGF-α-induced phosphorylation of AKT at 10 and 20 μM (Fig 5B).

### Effects of raloxifene and bazedoxifene on the TGF-α-induced phosphorylation of EGFR and AKT

**Fig 6:**
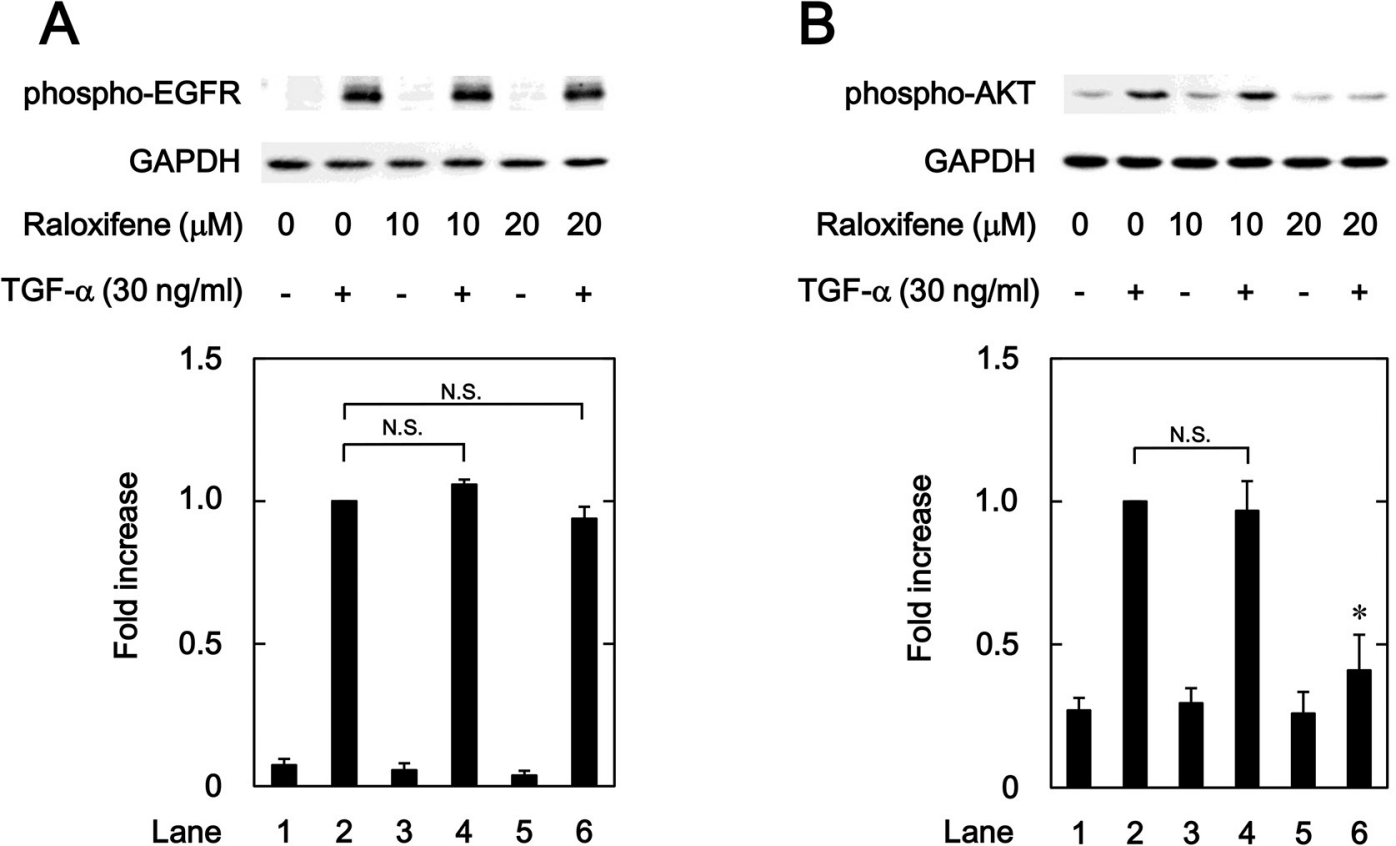
Effects of raloxifene on the TGF-α-induced phosphorylation of EGFR (A) and AKT (B) in HuH7 cells. The cells were pretreated with the indicated concentrations of raloxifene for 8 h, and then stimulated by 30 ng/ml of TGF-α or vehicle for 1 min. The histogram shows the quantitative representation of the phosphorylated levels after normalization with respect to GAPDH obtained from a densitometric analysis. The average of density levels of the TGF-α stimulation alone (lane 2) was expressed as 1.0. Each value represents the mean ± SD of triplicate determinations from three independent cell preparations. *p<0.05, compared to the value of the control cells with TGF-α stimulation alone (lane 2). N.S. designates no significant difference between the indicated pairs.

**Fig 7:**
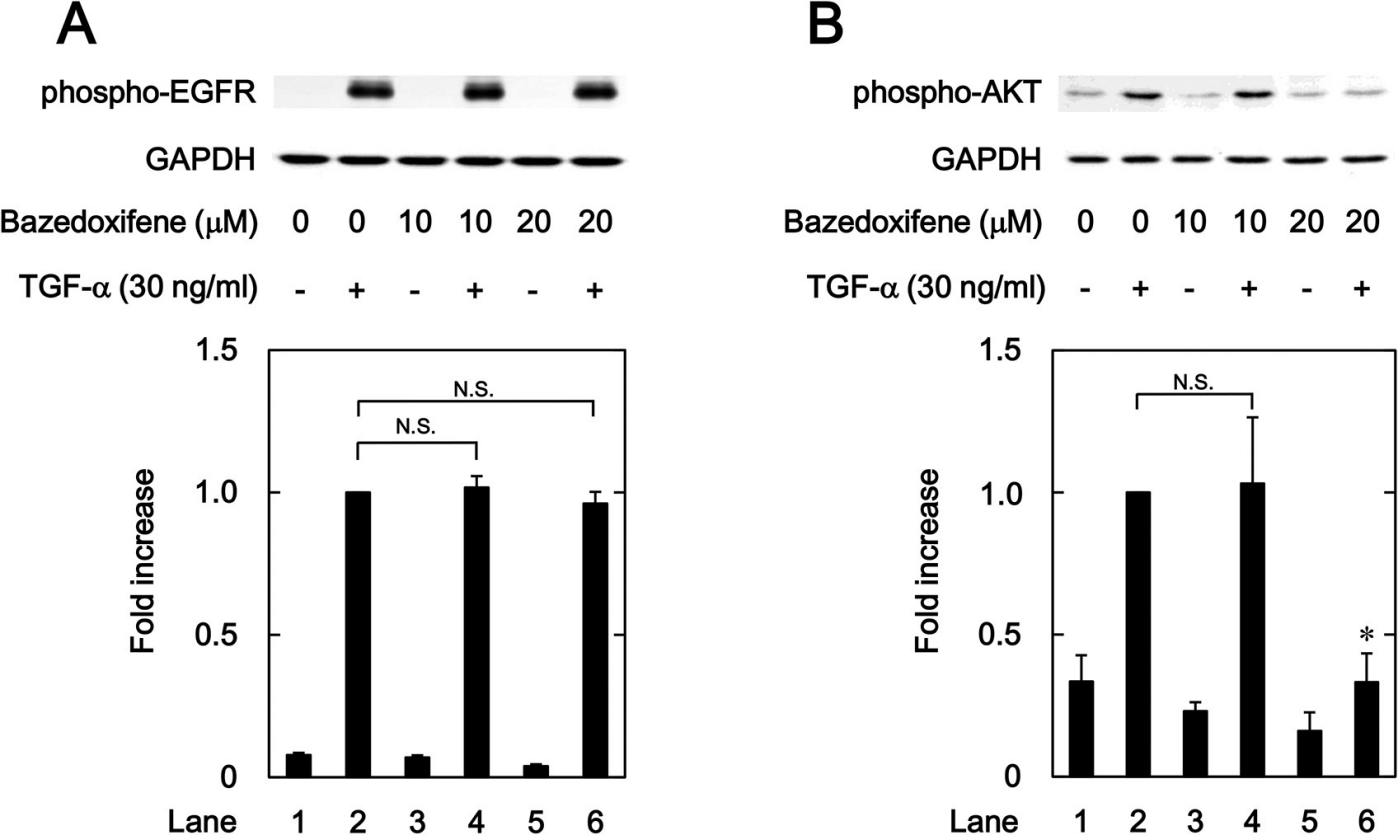
T Effects of bazedoxifene on the TGF-α-induced phosphorylation of EGFR (A) and AKT (B) in HuH7 cells. The cells were pretreated with the indicated concentrations of bazedoxifene for 8 h, and then stimulated by 30 ng/ml of TGF-α or vehicle for 1 min. The histogram shows the quantitative representation of the phosphorylated levels after normalization with respect to GAPDH obtained from a densitometric analysis. The average of density levels of the TGF-α stimulation alone (lane 2) was expressed as 1.0. Each value represents the mean ± SD of triplicate determinations from three independent cell preparations. *p<0.05, compared to the value of the control cells with TGF-α stimulation alone (lane 2). N.S. designates no significant difference between the indicated pairs.

We investigated whether raloxifene and bazedoxifene as well as ERβ agonists affect the TGF-α-induced AKT phosphorylation in HuH7 cells. Raloxifene, which had no effect on the TGF-α-induced auto-phosphorylation of EGFR (Fig 6A), significantly suppressed the phosphorylation of AKT by TGF-α (Fig 6B). Additionally, bazedoxifene markedly inhibited the TGF-α-induced phosphorylation of AKT without affecting the auto-phosphorylation of EGFR (Fig 7A and 7B).

### Effects of ERB041 and raloxifene on the AKT turnover

**Fig 8:**
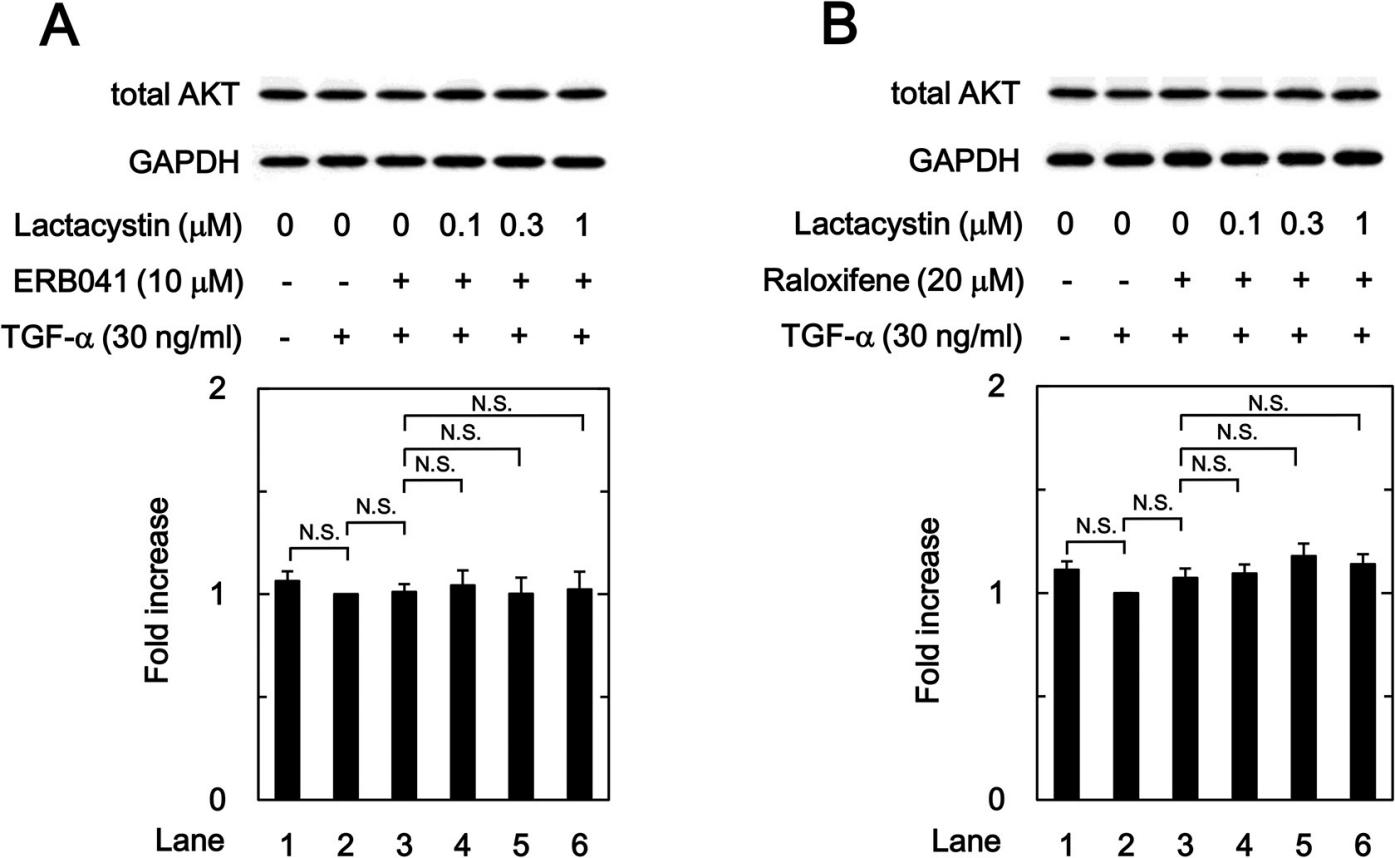
Effects of lactacystin on the amont of total AKT in the presence of ERB041 or raloxifene in HuH7 cells. The cells were treated with the indicated concentrations of lactacystin immediately prior to the pretreatment of 10 μM of ERB041 (A) or 20 μM of raloxifene (B) for 8h, and then stimulated by 30 ng/ml of TGF-α or vehicle for 1 min. The histogram shows the quantitative representation of the total AKT levels after normalization with respect to GAPDH obtained from a densitometric analysis. The average of density levels of the TGF-α stimulation alone (lane 2) was expressed as 1.0. Each value represents the mean ± SD of triplicate determinations from three independent cell preparations. N.S. designates no significant difference between the indicated pairs.

To verify the inhibitoion by the ERβ agonist and SERM of TGF-α-induced activation of AKT was not caused by effects on AKT turnover, we examined the effects of lactacystin [31], a proteasome inhibitor, on the amount of total AKT in the ERB041- or raloxifene-treated HuH7 cells. As shown in Fig 8, both ERB041 and raloxifene did not downregulate the amount of total AKT of HuH7 cells in the presence of TGF-α. In addition, lactacystin did not affect the amount of total AKT of TGF-α-stimulated HuH-7 cells in the presence of ERB041 or raloxifene. These results suggested that the suppressive effects of the ERβ agonists and SERMs on TGF-α-induced AKT activation might not be due to accelerated turnover of AKT.

### Effect of NSC23766 on the TGF-α-induced migration of HuH7 cells and the TGF-α-induced activation of Rac

**Fig 9:**
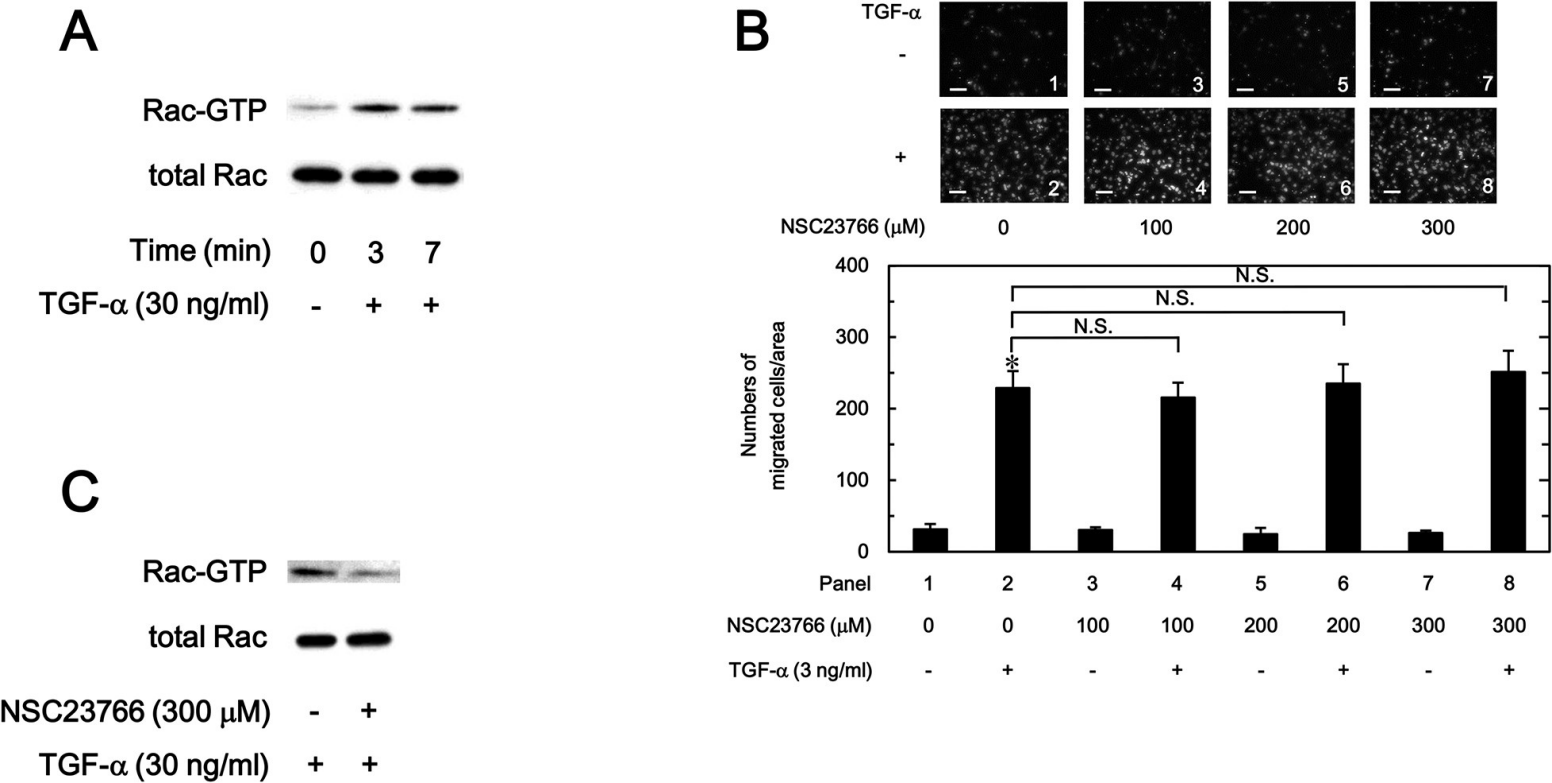
Effects of TGF-α and NSC23766 on the levels of GTP-binding Rac in HuH7 cells (A, C) and effect of NSC23766 on the TGF-α-induced migration of HuH7 cells (B). The cells were stimulated with 30 ng/ml of TGF-α for indicated times (A), or pretreated with 300 μM of NSC23766 or vehicle for 60 min, and then stimulated with 30 ng/ml of TGF-α for 5 min (C). The GTP-binding Rac in the cell extracts was immunoprecipitated using Rac1 activation assay kit. The immunoprecipitted GTP-binding Rac and pre-immunoprecipitated lysates (total Rac) were subjected to Western blot analysis using antibodies against Rac. (B), The cells were pretreated with indicated concentrations of NSC23766 for 60 min, and then stimulated by 3 ng/ml of TGF-α or vehicle for 23 h. The migrated cells were stained with DAPI for the nuclei. The cells were photographed by fluorescent microscopy at a magnification of 20× (upper panel) and counted (bar graph). Each value represents the mean ± SD of triplicate determinations from three independent cell preparations. *p<0.05, compared to the value of the control cells without TGF-α stimulation (panel 1). N.S. designates no significant difference between the indicated pairs. Scale bar: 100 μm.

Activation of Rac signaling pathway has been reportedly involved in HCC cell motility [32]. In addition, TGF-α is known to activate Rac [33, 34]. Therefore, we examined whether TGF-α activates Rac in the HuH7 cells. As shown in Fig 9A, TGF-α stimulated the Rac activity in HuH7 cells. However, NSC23766, a selective inhibitor of Rac1-GEF interaction [35], did not show inhibitory activity on TGF-α-induced migration of HuH7 cells even at a high concentration of 300 μM (Fig 9B). We additionally confirmed that 300 μM of NSC23766 inhibits TGF-α-induced Rac activation in HuH7 cells (Fig 9C). Therefore, suppressive effects on TGF-α-induced HuH7 cell migration of ERβ agonists might not correlate with Rac signaling pathway, and TGF-α-induced activity of Rac might be independent with the HuH7 cell migration.

### Effects of G-1 on the TGF-α-induced phosphorylation of AKT

**Fig 10:**
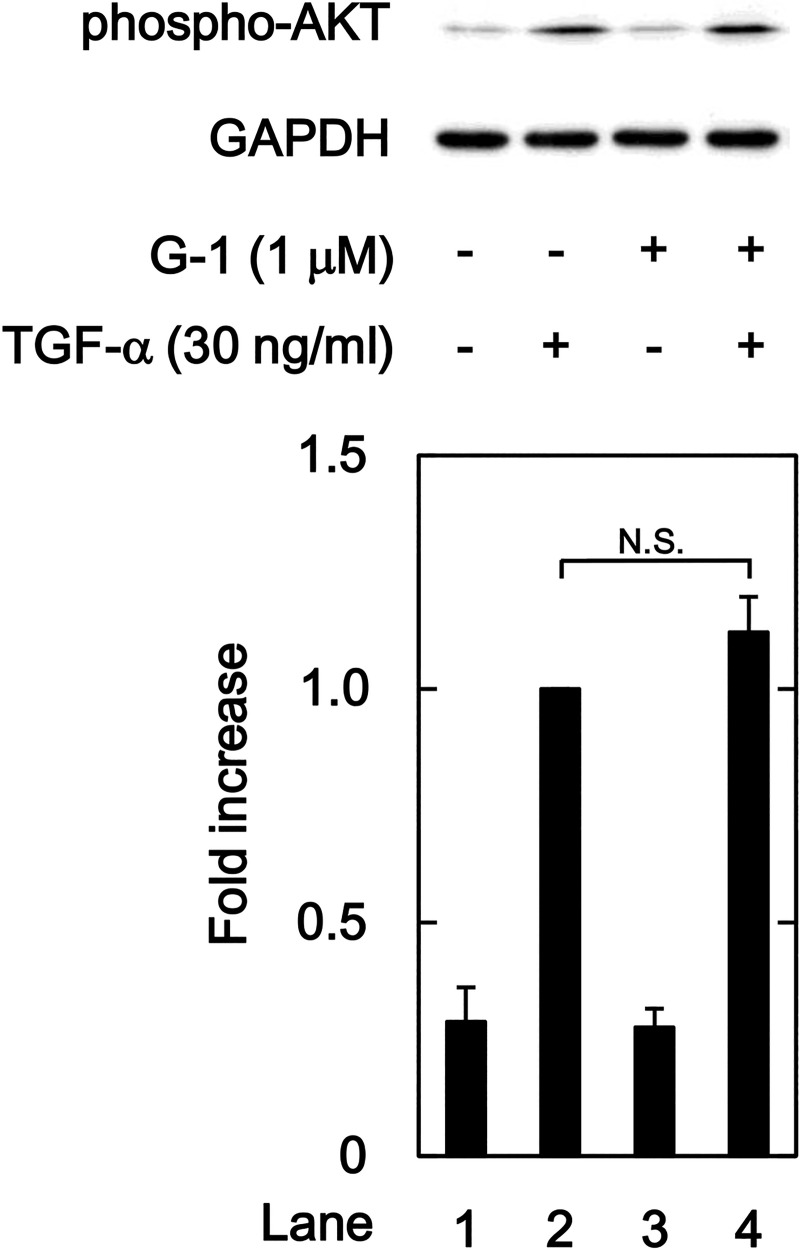
Effect of G-1 on the TGF-α-induced phosphorylation of AKT in HuH7 cells. The cells were pretreated with 1 μM of G-1 or vehicle for 1h and then stimulated by 30 ng/ml of TGF-α or vehicle for 1 min. The histogram shows the quantitative representation of the phospho-AKT levels after normalization with respect to GAPDH obtained from a densitometric analysis. The average of density levels of the TGF-α stimulation alone (lane 2) was expressed as 1.0. Each value represents the mean ± SD of triplicate determinations from three independent cell preparations. N.S. designates no significant difference between the indicated pairs.

Besides nuclear ERs, ERα and ERβ, a G protein-coupled ER, GPR30, was recently recognized as an ER that mediates non-genomic estrogen signaling [36]. Therefore, we examined the effects of G-1, an agonist of GPR30 [37], on the TGFα-induced activation of AKT in HuH7 cells to know whether an agonistic activity of SERM on GPR30 involves in the inhibition of TGFα-induced AKT activation. G-1 reportedly activates GPR30 in a human hepatoma cell, HuH7.5, at the concentrations up to 1 μM [38]. Thus, we examined the effect of G-1 on TGFα-induced activation of AKT in HuH7 cells at 1 μM. As shown in Fig 10, G-1 did not affect the TGFα-induced AKT activation in HuH7 cells. Agonistic activity of SERMs on GPR30 might not be involved in the suppression of TGFα-induced AKT activation in HuH7 cells.

### Effects of PHTPP on the suppression by raloxifene or bazedoxifene of the TGF-α-induced migration of HuH7 cells

**Fig 11:**
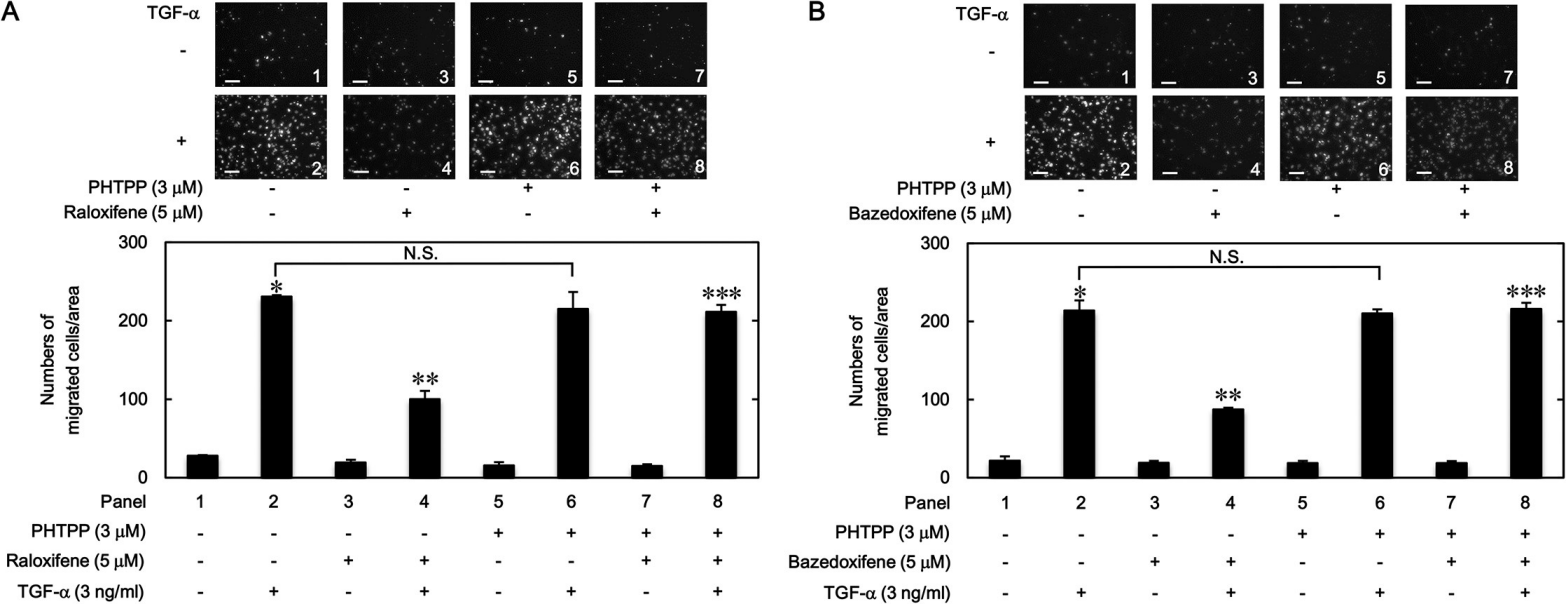
Effects of PHTPP on the suppression by raloxifene or bazedoxifene of the TGF-α-induced migration of HuH7 cells. The cells were exposed with 3 μM of PHTPP or vehicle for 60 min, and then pretreated with 5 μM of raloxifene (A), 5 μM of bazedoxifene (B) or vehicle for another 60 min. After the pretreatment, the cells were stimulated by 3 ng/ml of TGF-α or vehicle for 23 h. The migrated cells were stained with DAPI for the nuclei. The cells were photographed by fluorescent microscopy at a magnification of 20× (upper panel) and counted (bar graph). Each value represents the mean ± SD of triplicate determinations from three independent cell preparations. *p<0.05, compared to the value of the control cells without TGF-α stimulation (panel 1). **p<0.05, compared to the value of TGF-α stimulation alone (panel 2). ***p<0.05, compared to the value of TGF-α stimulation with the pretreatment of raloxifene or bazedoxifene (panel 4). N.S. designates no significant difference between the indicated pairs. Scale bar: 100 μm.

In order to furthermore investigate whether ERβ activated by raloxifene or bazedoxifene plays a suppressive role in the TGF-α-induced migration of HuH7 cells, we examined the effect of PHTPP, an antagonist of ERβ [29], on the cell migration. PHTPP significantly released the inhibition by raloxifene (Fig 11A) or bazedoxifene (Fig 11B) on the TGF-α-induced migration of HuH7 cells.

## Discussion

In the present study, we showed that raloxifene and bazedoxifene but not tamoxifen among SERM, suppressed the TGF-α-induced migration of HCC-derived HuH7 cells. It is generally known that ERs are classified into two subtypes, ERα and ERβ [1, 2], and that ERα and ERβ exist in HCC cells [15–17]. We next demonstrated that the activation of ERβ but not ERα leads to the inhibition of the HuH7 cell migration by TGF-α. Additionally, the suppressive effect of ERβ agonist on the cell migration was not affected by simultaneous stimulation of ERα agonist. By using PHTTP, an ERβ antagonist [29], we found that the suppressive activities of raloxifene and bazedoxifene on the TGF-α-induced migration of HuH7 cells were significantly blocked. Therefore, based on our findings, it is most likely that raloxifene and bazedoxifene suppress the TGF-α-induced migration of HuH7 cells via the activation of not ERα but ERβ.

The activity of SERM as an estrogen receptor regulator is target organ dependent, and SERM can play as both an agonist and an antagonist of ER [1–3]. Our present findings suggest that raloxifene and bazedoxifene might act as ERβ agonists in HCC-derived HCC cells. It has been reported that bazedoxifene (10 μM) alone suppresses the wound healing of HuH7 cells [39]. In the present study, we showed that 5 μM of bazedoxifene, which by itself failed to affect the HuH7 cell migration, significantly inhibited the TGF-α-induced cell migration. It is currently recognized that among growth factors, TGF-α closely correlates to HCC development [18–21]. As far as we know, this study is probably the first report showing that raloxifene and bazedoxifene have inhibitory activities on the TGF-α-induced migration of HCC cells. On the contrary, we demonstrated that tamoxifen, a SERM, failed to suppress the TGF-α-induced migration of HuH7 cells. Tamoxifen is a compound that has been developed as an ER antagonist for ERα positive breast cancer treatment [1–4] whereas tamoxifen acts as an agonist of ER in bone metabolism, resulting in the up-regulation of bone mass [1–4]. Regarding tamoxifen-effects on liver tissue, tamoxifen reportedly shows an ERα-dependent hepatoprotective effect against hepatotoxic agents [4]. In addition, it has been shown that tamoxifen induces steatosis in the liver [4], and mediates hepatocarcinogenesis by the formation of DNA adducts [2]. In HCC tumors, expression levels of ERα are markedly down-regulated compared to those in non-tumor liver tissue [15, 16], and ERα protein expression is also very low in HCC-derived HuH7 cells [17]. Taking our findings into account, it seems unlikely that tamoxifen plays as an agonist of ERβ, leading to the inhibition of the TGF-α-induced migration of HuH7 cells.

**Fig 12:**
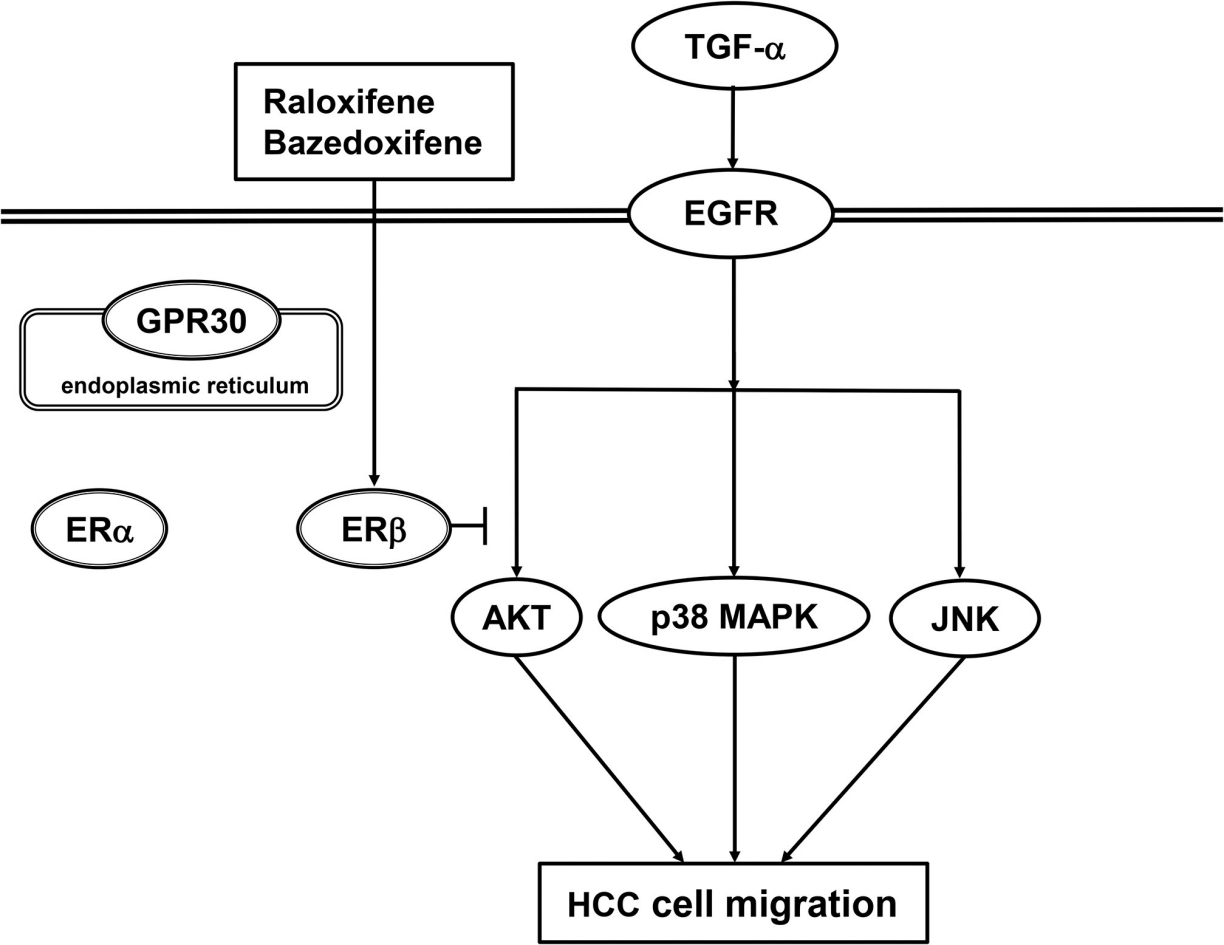
Schematic illustration of the mechanism behind the inhibition of the TGF-α-induced HCC cell migration by raloxifene and bazedoxifene. TGF, transforming growth factor; EGFR, epidermal growth factor receptor; ER, estrogen receptor; GPR30, G protein-coupled ER; MAPK, mitogen-activated protein kinase; JNK, c*-Jun* N-terminal kinase; HCC, hepatocellular carcinoma.

We have previously reported that p38 MAPK, JNK and AKT act as positive regulators in the TGF-α-induced migration of HuH7 cells [23, 24]. In the present study, we showed that ER041 and DNP, agonists of ERβ, did not affect the TGF-α-induced EGFR auto-phosphorylation. In addition, raloxifene and bazedoxifene failed to affect the auto-phosphorylation of EGFR. Thus, it seems unlikely that the activation of ERβ leads to the suppression of EGFR activated by TGF-α. Next, we demonstrated that ERB041 markedly reduced the TGF-α-induced phosphorylation of AKT without affecting the phosphorylation of p38 MAPK or JNK. Furthermore, DPN, raloxifene and bazedoxifene also showed inhibitory effects on the TGF-α-induced AKT phosphorylation. Therefore, our results suggest that the TGF-α-induced activation of AKT, but not p38 MAPK and JNK, is suppressed by activated ERβ in HuH7 cells. Based on our findings as a whole, it is most likely that raloxifene and bazedoxifene reduce the TGF-α-induced migration of HCC-derived HuH7 cells mediated through inhibiting the AKT signaling pathway via ERβ. The potential mechanism underlying inhibition by raloxifene and bazedoxifene of the TGF-α-induced migration of HCC cells is summarized in Fig 12.

It is generally established that raloxifene and bazedoxifene act as agonists of ER in bone, and antagonists of ER in uterus and breast [2, 4]. Currently, raloxifene and bazedoxifene are approved for the treatment of postmenopausal osteoporosis with favorable effects on uterine and breast tissue, and used clinically worldwide [40]. ERα and ERβ are expressed equally levels in breast, bone and uters [2]. While, high levels of ERα is expressed in hepatocytes [2]. Although espression of ERα is high in hepatocytes, it has been reported that ERα36, an ERα splicing variant lacking a ligand-binding domain, is predominantly expressed in HCC cells, and sometimes becomes the only form of ERα [16, 41, 42]. Therefore, SERMs might not interact with ERα, and only ERβ mediated effect might be expressed in HCC cells. It has been reported that tamoxifen resistance is correlated with increased expression of ERα36 in breast cancer [43] and ERβ expression reduces cell mobility in breast and ovarian cancers [44]. Thus, the activity of SERMs as ERβ agonists might contribute to suppressing the progression of cancer not only in the liver but also in other tissues, such as the breast. Our results show that the activation of ERβ inhibits the migrating activity of HCC cells provides the basis for a potential new aspect of preventing HCC metastasis. SERM including raloxifene and bazedoxifene with ERβ agonistic activities might be potent therapeutic candidates for the therapy of HCC, especially for preventing metastasis. Further investigations are needed to clarify the exact roles of SERM and the mechanism of ERβ in the migration of HCC cells.

In conclusion, our results strongly suggest that raloxifene and bazedoxifene suppress the TGF-α-induced cell migration of HCC through ERβ-mediated inhibition of the AKT signaling pathway.

## Supporting information

S1 Raw images Original uncropped and unadjusted images of Fig 4. (PDF)

S2 Raw images Original uncropped and unadjusted images of Fig 5. (PDF)

S3 Raw images Original uncropped and unadjusted images of Fig 6. (PDF)

S4 Raw images Original uncropped and unadjusted images of Fig 7. (PDF)

S5 Raw images Original uncropped and unadjusted images of Fig 8. (PDF)

S6 Raw images Original uncropped and unadjusted images of Fig 9. (PDF)

S7 Raw images Original uncropped and unadjusted images of Fig 10. (PDF)
