# Supplementary figures and images for: SERMs (selective estrogen receptor modulator), acting as estrogen receptor β agonists in hepatocellular carcinoma cells, inhibit the transforming growth factor-α-induced migration via specific inhibition of AKT signaling pathway
Source: PLoS One. 2022 Jan 10;17(1):e0262485. doi: 10.1371/journal.pone.0262485 (PMC8746762; doi:10.1371/journal.pone.0262485)

S1 Fig.

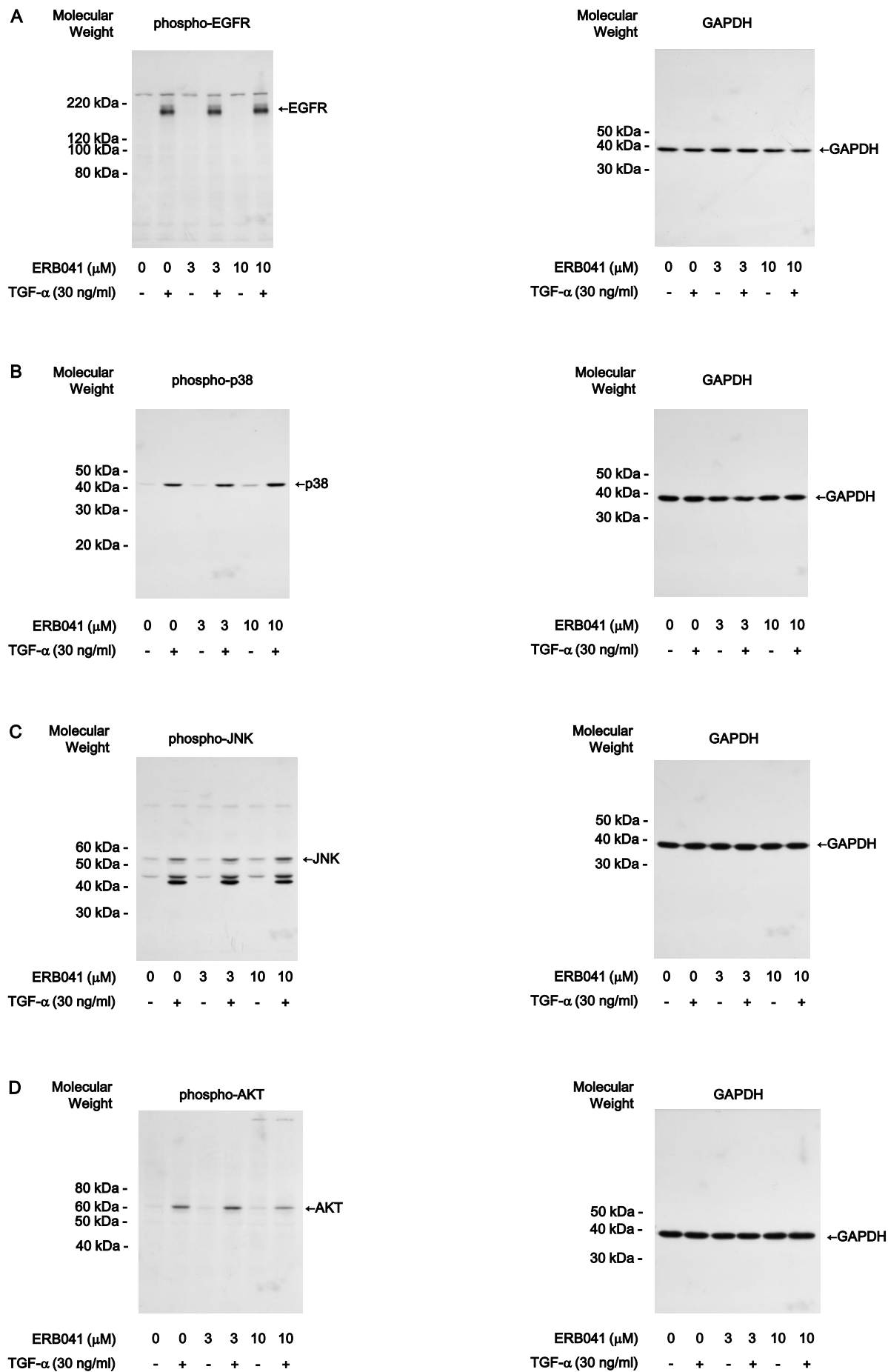

Supplement: S1 Raw images — (PDF) [file pone.0262485.s001.pdf]

S2 Fig.

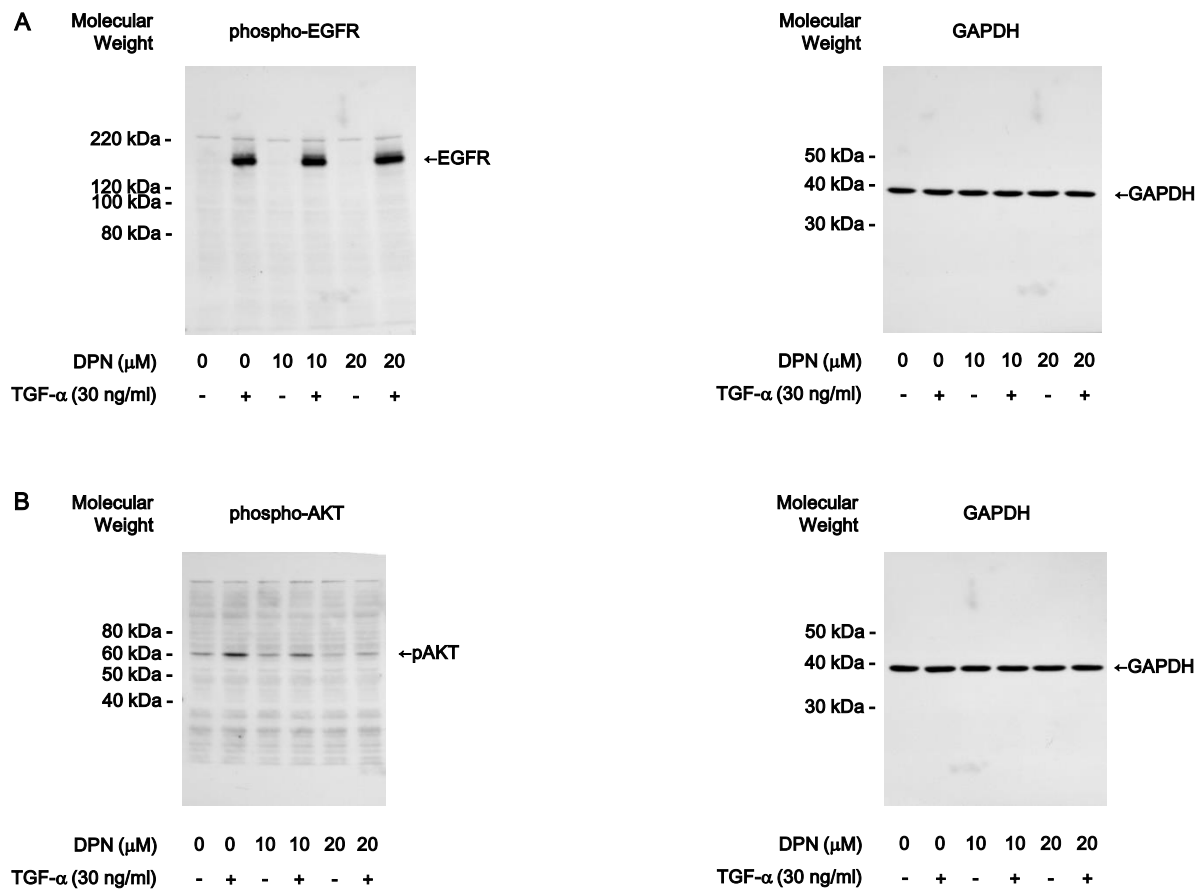

Supplement: S2 Raw images — (PDF) [file pone.0262485.s002.pdf]

S3 Fig.

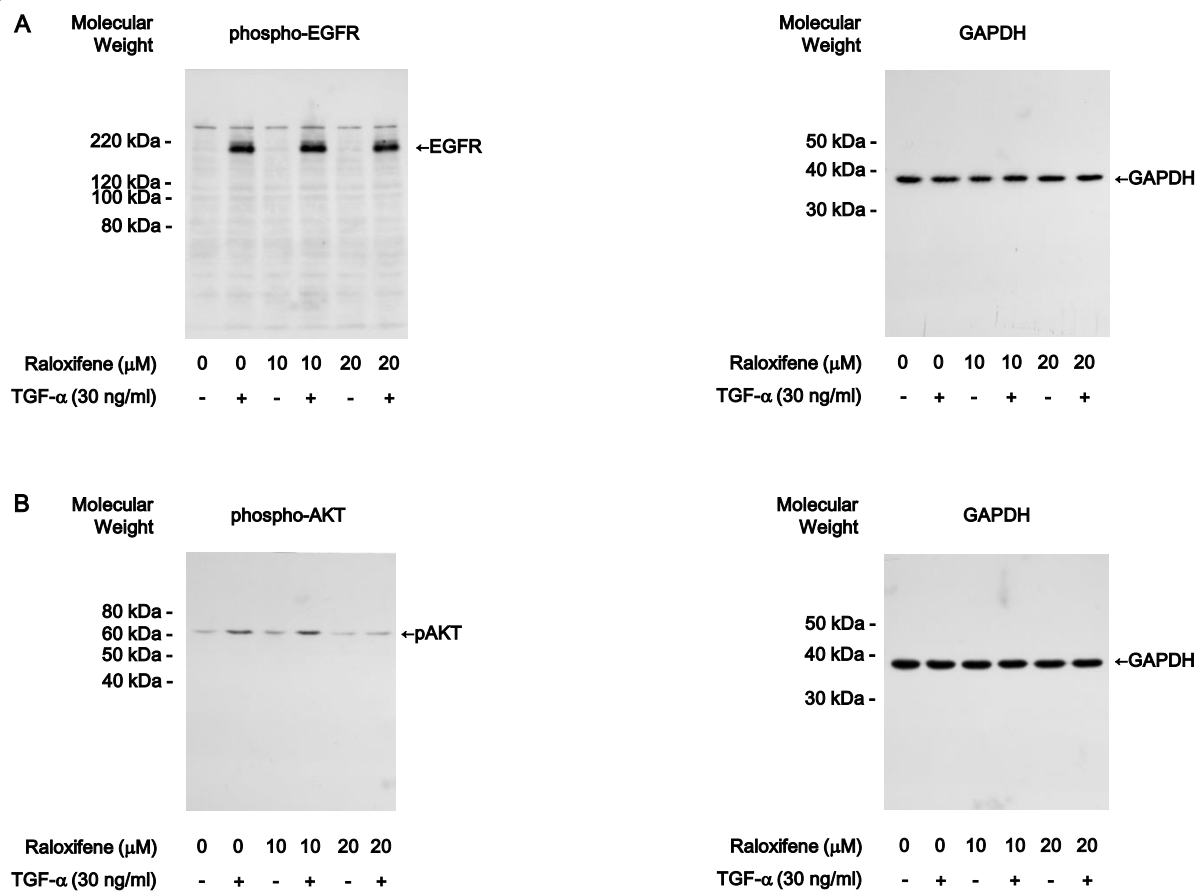

Supplement: S3 Raw images — (PDF) [file pone.0262485.s003.pdf]

S4 Fig.

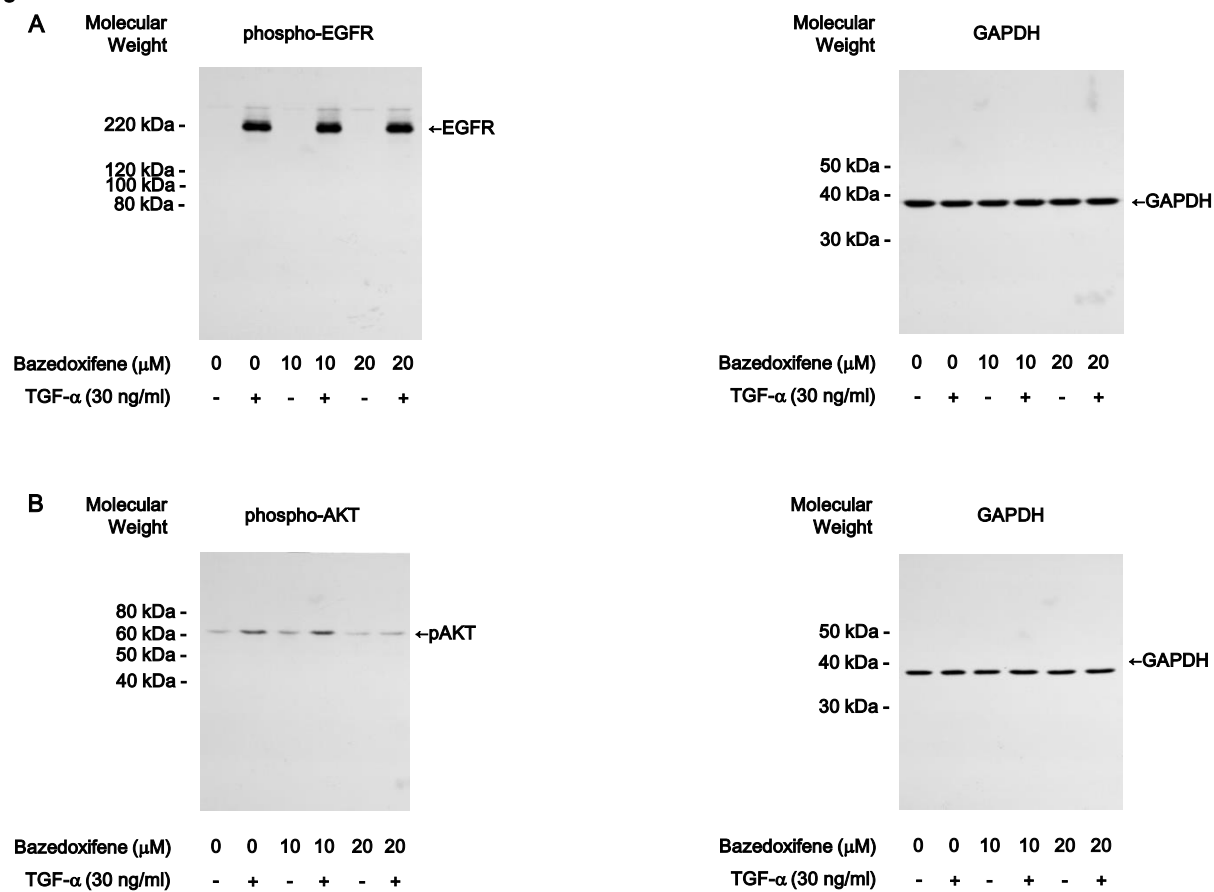

Supplement: S4 Raw images — (PDF) [file pone.0262485.s004.pdf]

S5 Fig.

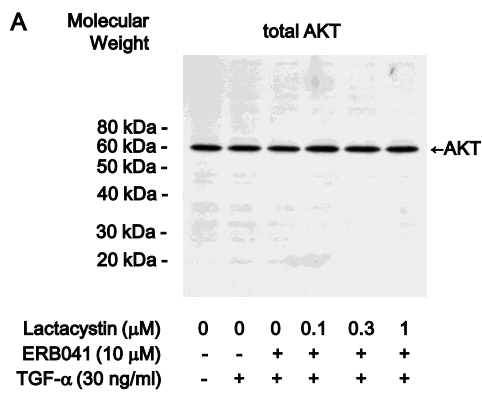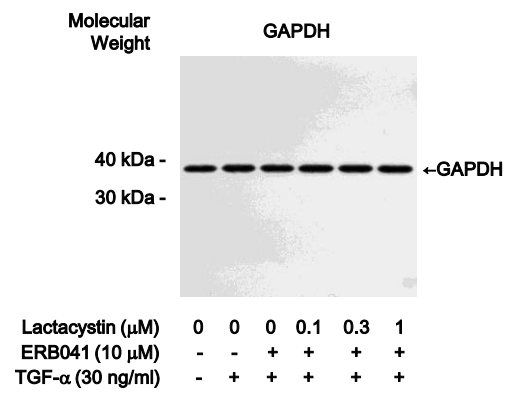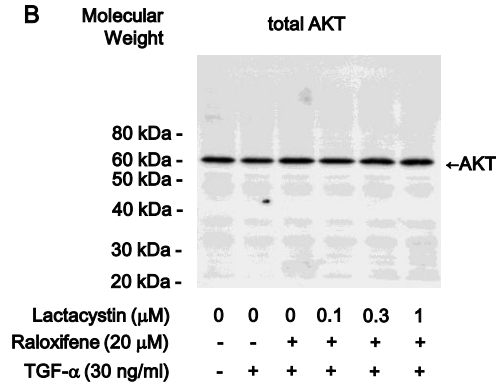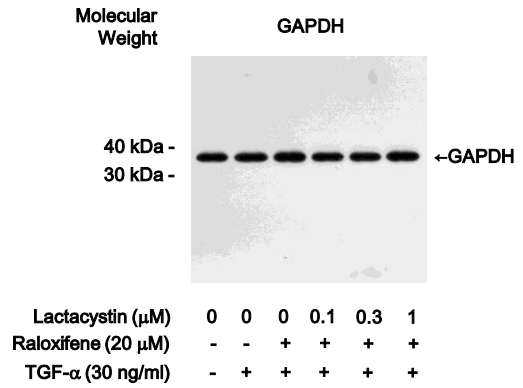

Supplement: S5 Raw images — (PDF) [file pone.0262485.s005.pdf]

S6 Fig.

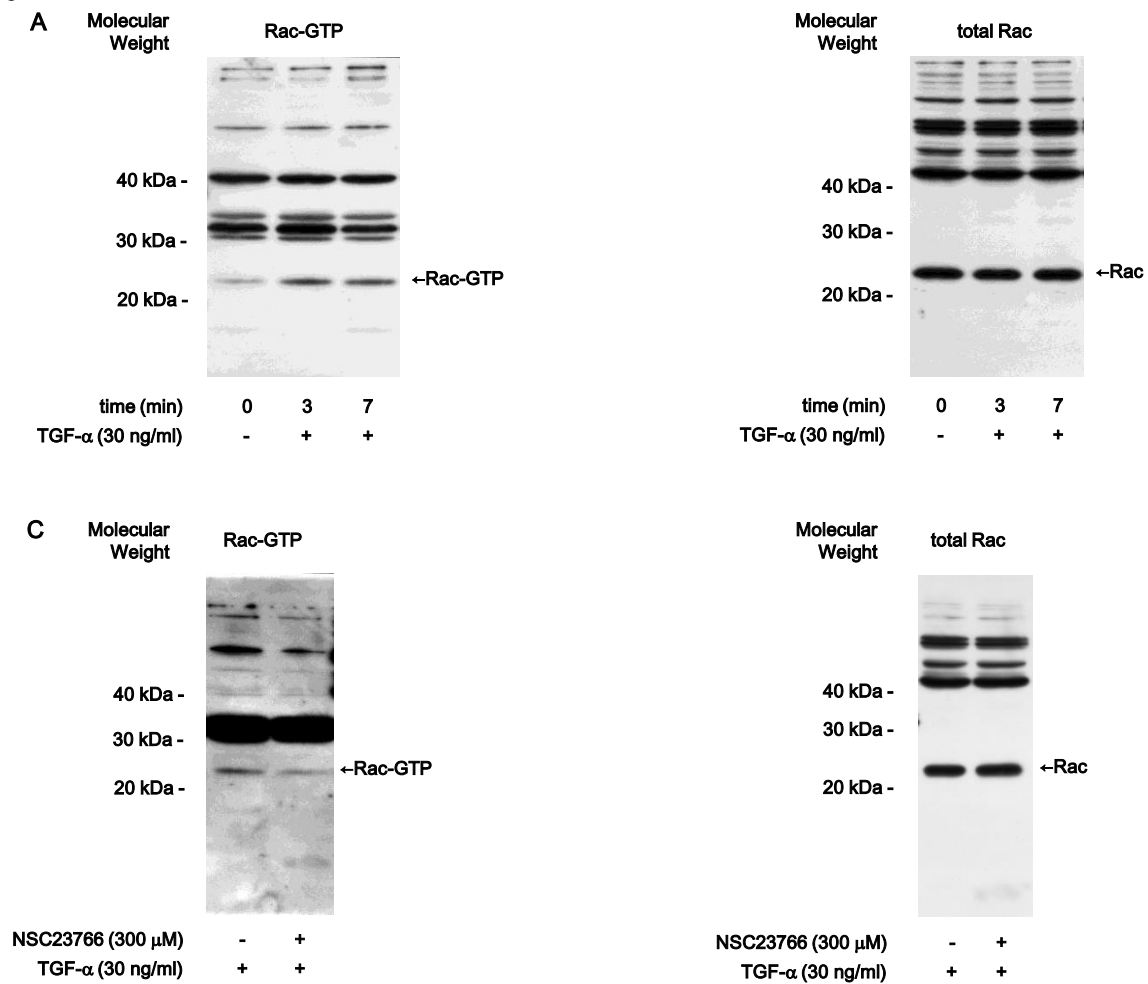

Supplement: S6 Raw images — (PDF) [file pone.0262485.s006.pdf]

S7 Fig.

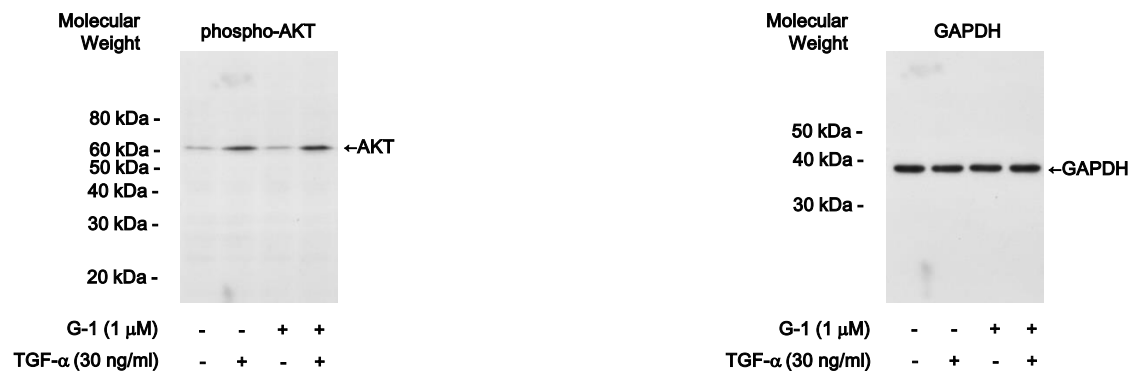

Supplement: S7 Raw images — (PDF) [file pone.0262485.s007.pdf]
